# Modelling the electric field in reactors yielding cold atmospheric–pressure plasma jets

**DOI:** 10.1038/s41598-020-61939-7

**Published:** 2020-03-30

**Authors:** P. Vafeas, P. K. Papadopoulos, G. P. Vafakos, P. Svarnas, M. Doschoris

**Affiliations:** 1 Department of Chemical Engineering, University of Patras, 26504 Patras, Greece; 2 Department of Mechanical Engineering and Aeronautics, University of Patras, 26504 Patras, Greece; 3 Department of Electrical and Computer Engineering, High Voltage Laboratory, University of Patras, 26504 Patras, Greece; 4 Institute of Genetics and Biometry, Leibniz Institute for Farm Animal Biology, 18196 Dummerstorf, Germany

**Keywords:** Applied mathematics, Plasma physics

## Abstract

The behavior of the electric field in Cold Atmospheric–Pressure Plasma jets (CAPP jets) is important in many applications related to fundamental science and engineering, since it provides crucial information related to the characteristics of plasma. To this end, this study is focused on the analytic computation of the electric field in a standard plasma reactor system (in the absence of any space charge), considering the two principal configurations of either one–electrode or two–electrodes around a dielectric tube. The latter is considered of minor contribution to the field calculation that embodies the working gas, being an assumption for the current research. Our analytical technique employs the cylindrical geometry, properly adjusted to the plasma jet system, whereas handy subdomains separate the area of electric activity. Henceforth, we adapt the classical Maxwell’s potential theory for the calculation of the electric field, wherein standard Laplace’s equations are solved, supplemented by the appropriate boundary conditions and the limiting conduct at the exit of the nozzle. The theoretical approach matches the expected physics and captures the corresponding essential features in a fully three–dimensional fashion via the derivation of closed–form expressions for the related electrostatic fields as infinite series expansions of cylindrical harmonic eigenfunctions. The feasibility of our method for both cases of the described experimental setup is eventually demonstrated by efficiently incorporating the necessary numerical implementation of the obtained formulae. The analytical model is benchmarked against reported numerical results, whereas discrepancies are commented and prospective work is discussed.

## Introduction

Cold Atmospheric Pressure Plasma jets (CAPP jets) are a hot research topic due their potentiality in many biomedical applications^1^. Indicatively, we refer sterilization^2^, treatment of tissues^3^ and liposomes^4,5^, as well as cells including cancer treatment^6^. The study of CAPP jets is based on experiments that aim to characterize the plasma by providing information on the ionic species, the properties of the plasma plume, its temperature, etc.^7–10^, and on numerical studies that implement various microscopic and macroscopic models in order to simulate CAPP jets^11–14^.

One important parameter in the study of CAPP jets is the electric field in the plasma reactor. The geometry of the electrodes and the waveform features of the applied voltage affect the ignition of the plasma and the discharge dynamics, as well as the characteristics of the plasma plume^15,16^. The electric field in the electrode region is usually examined via numerical simulations, which focus on the dynamics of the plasma. However, there are a few researches that examine the effect of the plasma reactor configuration on the electric field, such as^15^ and^17^, wherein the authors study numerically the effect of the dielectric tube radius and of the relative permittivity of the dielectric material on the electric field and the propagation velocity of the discharge front.

However, limitation may be arisen from purely numerical solutions. A possible one, in the numerical approaches that simulate plasma either macroscopically or microscopically, is the inability to impose the boundary condition for the vanishing electrostatic potential at infinity^18^. This may be critical in simulations that involve reactors with a single driven electrode, where the role of the grounded electrode is played by the far–field. This problem can be tackled in the finite solution domain of the numerical simulations, by using the Neumann to Dirichlet artificial boundary conditions^19^ or it can be circumvented by considering a grounded surface at the limits of the computational domain^15^. Analytical solutions do not have such limitations and can be very useful in the calculation of potentials in various engineering applications. For instance, in^20,21^ electrostatically actuated microelectromechanical (MEMS) and nanoelectromechanical systems (NEMS) are investigated, while in^22^ the authors examine the electrostatic potential of a point charge embedded in a three–layered dielectric system with infinite planar interfaces using the technique of Hankel transform.

In the present work, we develop an analytical solution of the electric field for two of very commonly employed plasma–jet reactor configurations. The first plasma reactor consists of a dielectric tube with a single outer electrically biased electrode; the second consists of a dielectric tube with two outer electrodes, one of which is electrically biased and the other one is grounded. The motivation for this paper is to obtain analytical solutions for the electric field that can be employed in plasma simulations, instead of time consuming numerical solutions. These solutions provide an easy means of estimating the spatial distribution of the electric field for different geometric characteristics, i.e. electrode lengths and gap distances, which are important factors for the properties of the plasma. Furthermore, new physical results can be derived from analytical techniques and they can be used even for the verification of numerical results. Therefore, there is always room for such kind of methods that co–exist with pure numerical codes and aim to the solution of boundary value problems in physical applications of importance. Under this aspect, this article has endeavored to show that an analytical approach to problems involving CAPP jets is conceivable.

The methodology for the analytical solutions is primarily based on the selection of the best fitted cylindrical coordinate system^23^ for the current investigation, conveniently accommodated to the plasma reactor system for modelling purposes. Therein, the domain of field activity, suitably divided into subsectors, is localized in the working gas, whereas the dielectric tube’s effect is not taken into account in order to facilitate the derivation of the analytical solution. Otherwise, the electrostatic limit of Maxwell’s equations^24^ is considered in such cases, thus the electrostatic potentials within each separate area, which are created by the electrode configurations in both cases, are governed by the Laplace’s equation. Hence, they are expanded in terms of cylindrical harmonic eigenfunctions^25^, since the corresponding electric fields, produced by the gradient of the pre–mentioned harmonic potentials, are divergence–free. Then, the appropriate boundary and limiting at infinity (far away from the exit of the jet) conditions are implied, in order to obtain a sequence of well–posed boundary value problems, which are readily solved and the implicated fields are provided in a compact fashion via closed–type series expansions. Recollecting the key features of the aforementioned analysis, including the indispensable physical and geometrical parameters, the analytical results are obtained through the requisite numerical implementation, showing accordance with the expected electric field behavior. The analytical model is eventually benchmarked against reported numerical results, whereas discrepancies are commented and prospective work is discussed.

## Problem Formulation and Mathematical Modeling

**Figure 1:**
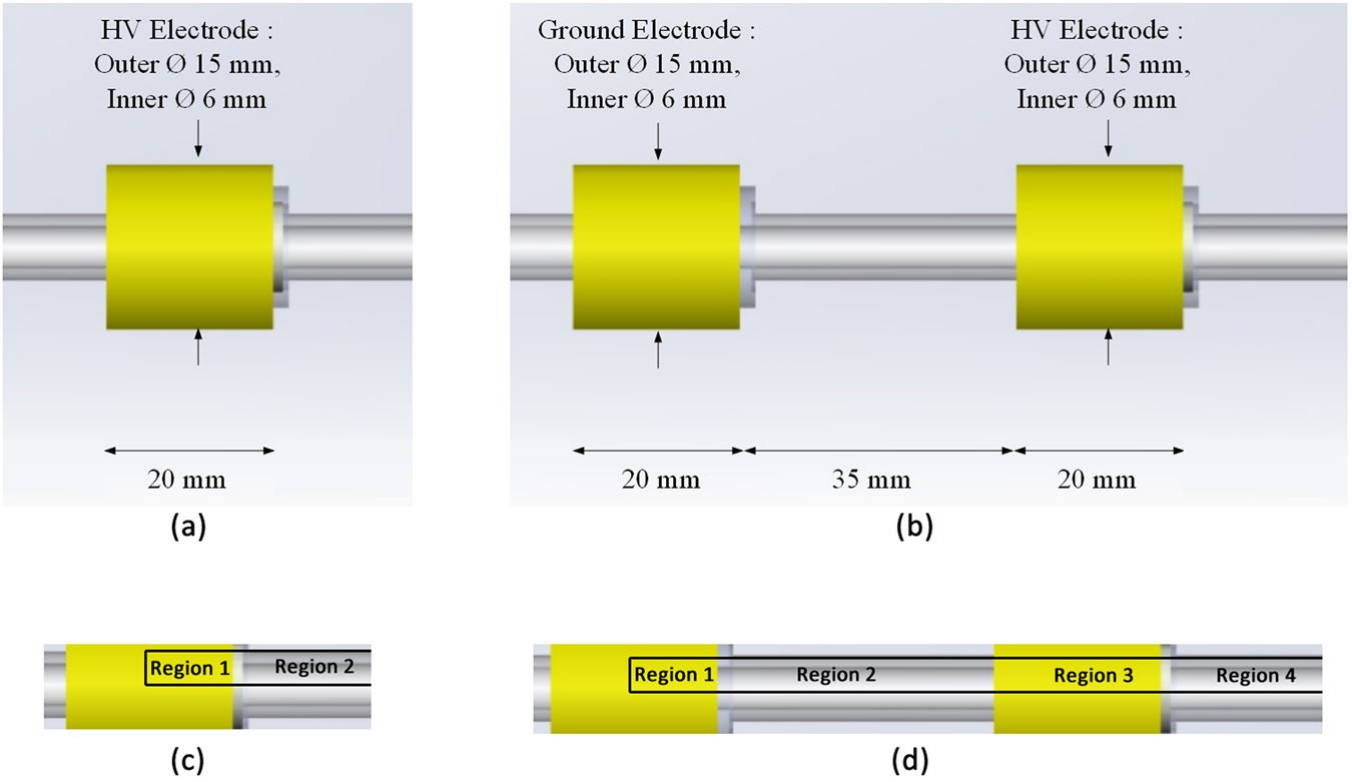
Plasma reactors with (**a**) a single external electrode and (**b**) two external electrodes. The computed regions for each reactor are shown in (**c**,**d**), respectively.

The experimental setup of the two plasma reactors that we consider is shown in Fig. 1. Both reactors have an electrode that is electrically biased, to create the necessary electric field for the initiation of the electrical discharges. In the plasma–jet applications the applied field is usually time–varying, where the waveform might be sinusoidal or pulsed. As a first step, in the present work we consider a steady state problem that corresponds to the beginning of the pulse in a pulsed voltage waveform. Moreover, at this stage and for modelling purposes, we neglect the effect of the dielectric material of the dielectric tube by considering only the electric permittivity of the working gas. This is expected to affect the results by predicting a higher peak value of the electrostatic potential close to the electrodes and a faster descent away from them. To assess the effect of this assumption on the results, we examine below the numerical simulation of the electric field in Jánský and Bourdon^15^, where it can be seen that the discrepancy in the axial distribution of the electric field for a dielectric tube with relative permittivity ${\varepsilon }_{r}=4$ is relatively small. It is noted that this is not the case in general, as the effect of the dielectric on the electric field is more pronounced in the radial component. Viegas *et al*.^26^ showed that the axial component of the electric field undergoes a small change in the rate of decrement, across the dielectric tube. The radial component, on the other hand is significantly affected, especially in the presence of a charge density, during the streamer propagation. Thus, in the present work, we consider that the assumption of a negligible dielectric, which is necessary for the analytical solution, is a fair conjecture for the electrostatic field.

**Figure 2:**
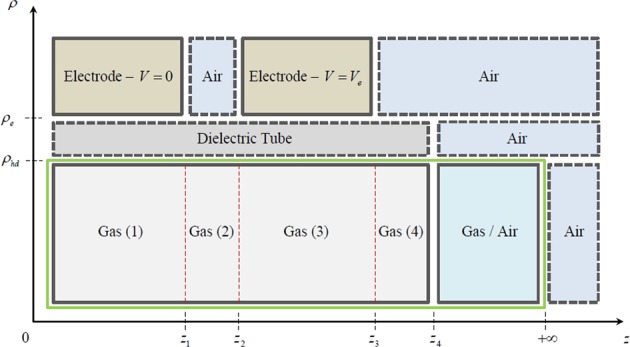
Plasma reactor cross–section and the distinguished areas of electrostatic activity.

The two plasma reactors that we consider are based on the experimental configuration that is very common in the plasma–jet community^27–29^. The first reactor (Fig. 1(a)) consists of an annular electrode placed around a dielectric tube. The solution area consists of two regions, as indicated in Fig. 1(c), which include the cylindrical area inside the electrode and extend from the half–length of the electrode on the left to infinite distance on the right. The second reactor (Fig. 1(b)) consists of two annular electrodes placed around a dielectric tube, the first of which is grounded and the second is electrically biased. The solution domain in this case consists of four regions, as indicated in Fig. 1(d), which include the cylindrical area inside the electrodes and extend from the half–length of the electrode on the left to infinite distance on the right. Aiming to mathematically model the electrostatic activity within the plasma reactor system (Fig. 1), we discriminate it into properly adjusted subsectors as demonstrated in Fig. 2 (this scheme incorporates both configurations, with the first one to be a subcase of the second). In this concept, air is treated as a single species, so a binary gas–air fluid system is considered. To this end, we implement to our work the circular cylindrical coordinate system^23^, which in terms of the variables $\rho \in [0,+\,\infty )$, $\varphi \in [0,2\pi )$ and $z\in (-\infty ,+\,\infty )$, it is defined via either the Cartesian or the cylindrical basis as

1 $${\bf{r}}=\mathop{\sum }\limits_{i=1}^{3}{x}_{i}{\hat{{\boldsymbol{x}}}}_{i}=\rho \hat{{\boldsymbol{\rho }}}+z\hat{{\boldsymbol{z}}},\,{\rm{where}}\,{x}_{1}=z,\,{x}_{2}=\rho \,\cos \,\varphi \,{\rm{and}}\,{x}_{3}=\rho \,\sin \,\varphi ,$$

in view of the position vector **r**, while

2 $$\hat{{\boldsymbol{\rho }}}=\,\cos \,\varphi {\hat{{\boldsymbol{x}}}}_{2}+\,\sin \,\varphi {\hat{{\boldsymbol{x}}}}_{3},\,\hat{{\boldsymbol{\varphi }}}=-\,\sin \,\varphi {\hat{{\boldsymbol{x}}}}_{2}+\,\cos \,\varphi {\hat{{\boldsymbol{x}}}}_{3}\,{\rm{and}}\,\hat{{\boldsymbol{z}}}={\hat{{\boldsymbol{x}}}}_{1}$$

denote the corresponding unit coordinate vectors of the system. Our problem is adjusted to the type of circular cylindrical geometry introduced here, where the ${x}_{1}$–axis is the axis of symmetry of an infinite circular cylinder and the other two axis are located properly so as to obtain a $(\rho ,\varphi ,z)$ clockwise system. More specific, the cylindrical system of coordinates is geometrically set in such a way so as the *z*–axis to coincide with the axis of symmetry of the plasma reactor system, while the axis corresponding to the $\rho$–variable to intersect it vertically for every $\varphi \in [0,2\pi )$. Therein, *z*_1_ and ${z}_{3}-{z}_{2}$ stand for the lengths of the grounded electrode with a vanishing potential and the electrode comprising a constant potential ${V}_{e}$, respectively, both attached to the dielectric tube of length ${z}_{4}$ at $\rho ={\rho }_{e}$, whereas air occupies the area between the two electrodes for ${z}_{1} < z < {z}_{2}$. The interface that separates the dielectric tube from the main gas flow is set at $\rho ={\rho }_{hd}$, while at the exit of the system at $z={z}_{4}$ and for $\rho \in [0,{\rho }_{hd}]$, we attain a mixture of gas and air flow. Obviously, moving far away towards $z\to +\,\infty$ for any $\rho \in [0,{\rho }_{e}]$, there is no electrostatic activity, leading to a zero potential, thus zero electric field.

A crucial remark related to the modelling analysis of this work is the assumption according to which the quartz tube does not contribute to the electric field distribution. In other words, we neglect the effect of the dielectric tube by considering only the electric permittivity of the working gas, as explained earlier. Consequently, the potential differential change within the dielectric tube is negligible, implying zero electric field for every ${\rho }_{hd} < \rho  < {\rho }_{e}$ and $z\in [0,{z}_{4}]$. Henceforth, the dielectric tube is not considered in the current model and, therefore, the two electrodes have a direct effect upon the working gas flow at $\rho ={\rho }_{hd}$ due to the presence of the externally controlled conditions. By virtue of this simplification, the area of electrostatic activity in our model is confined by the green frame in Fig. 2, while the bounded domains of the working gas, separated by four distinct areas and the area of the mixture of gas and air flow, are

3 $${\Omega }_{1}=\{{\bf{r}}\equiv (\rho ,\varphi ,z)\in {{\mathbb{R}}}^{3}:\rho \in (0,{\rho }_{hd}),\varphi \in [0,2\pi ),z\in (0,{z}_{1})\},$$

4 $${\Omega }_{2}=\{{\bf{r}}\equiv (\rho ,\varphi ,z)\in {{\mathbb{R}}}^{3}:\rho \in (0,{\rho }_{hd}),\varphi \in [0,2\pi ),z\in ({z}_{1},{z}_{2})\},$$

5 $${\Omega }_{3}=\{{\bf{r}}\equiv (\rho ,\varphi ,z)\in {{\mathbb{R}}}^{3}:\rho \in (0,{\rho }_{hd}),\varphi \in [0,2\pi ),z\in ({z}_{2},{z}_{3})\},$$

6 $${\Omega }_{4}=\{{\bf{r}}\equiv (\rho ,\varphi ,z)\in {{\mathbb{R}}}^{3}:\rho \in (0,{\rho }_{hd}),\varphi \in [0,2\pi ),z\in ({z}_{3},{z}_{4})\}$$

and

7 $${\Omega }_{m}=\{{\bf{r}}\equiv (\rho ,\varphi ,z)\in {{\mathbb{R}}}^{3}:\rho \in (0,{\rho }_{hd}),\varphi \in [0,2\pi ),z\in ({z}_{4},+\infty )\},$$

in which, by definition of the gradient $\nabla$ and the Laplacian Δ differential operators

8 $$\nabla =\hat{{\boldsymbol{\rho }}}\frac{\partial }{\partial \rho }+\frac{\hat{{\boldsymbol{\varphi }}}}{\rho }\frac{\partial }{\partial \varphi }+\hat{{\boldsymbol{z}}}\frac{\partial }{\partial z}\,{\rm{and}}\,\Delta =\frac{1}{\rho }\frac{\partial }{\partial \rho }\left(\rho \frac{\partial }{\partial \rho }\right)+\frac{1}{{\rho }^{2}}\frac{{\partial }^{2}}{\partial {\varphi }^{2}}+\frac{{\partial }^{2}}{\partial {z}^{2}}$$

for any $\rho \in [0,+\,\infty )$, $\varphi \in [0,2\pi )$ and $z\in (-\infty ,+\,\infty )$, Maxwell’s equations^24^ reduce to

9 $$\Delta {V}_{j}({\bf{r}})=0,\,{\rm{since}}\,{{\bf{E}}}_{j}({\bf{r}})=-\,\nabla {V}_{j}({\bf{r}})\,{\rm{and}}\,\nabla \cdot {{\bf{E}}}_{j}({\bf{r}})=0\,{\rm{with}}\,j=1,2,3,4,m$$

for ${\bf{r}}\in {\Omega }_{j}$, where *V*_*j*_ and **E**_*j*_ for $j=1,2,3,4,m$ are the corresponding to each region (3)–(7) electrostatic potentials and electric fields, respectively.

At this point, the system modeling has been hatched and an analytical solution will be presented. Though, it should be underlined that: The characteristics of common potential theory, incorporating harmonic functions as in (9), are well established and standard techniques have been developed when the domain boundaries involve a certain kind of conditions, for instance either Dirichlet or Neumann type. However, the situation becomes complicated when the solution of the given equation is required to satisfy different boundary conditions on disjoint parts of the boundary of the domain where the condition is stated^30^, as it is the case of the current research. Several mathematical methods have been proposed in manipulating such physical problems, indicating that there are no straightforward techniques towards the representation via certain solutions^30^. Hence, here we are obliged to solve several mixed–type boundary value problems, wherein the given solution of the Laplace’s Eq. (9) is based upon the classical method of separation of variables and the produced cylindrical harmonic eigenfunctions. Additional terms in this main solution could be incorporated in future work as an optimization process.

On the other hand, due to the rotational symmetry and without loss of generality, we may proceed to our analysis retaining azimuthal independence and consequently to exclude the $\varphi$–dependence in any potential field–calculation. Under this aspect, the electrostatic potential functions admit

10 $${V}_{1}({\bf{r}})={V}_{1,c}+\mathop{\sum }\limits_{n=1}^{+\infty }{J}_{0}({\lambda }_{n}^{(1)}\rho )[{A}_{n}^{(1)}\exp ({\lambda }_{n}^{(1)}z)+{B}_{n}^{(1)}\exp (-{\lambda }_{n}^{(1)}z)]\,{\rm{for}}\,{\bf{r}}\in {\Omega }_{1},$$

11 $${V}_{2}({\bf{r}})={V}_{2,c}+\mathop{\sum }\limits_{n=1}^{+\infty }{J}_{0}({\lambda }_{n}^{(2)}\rho )[{A}_{n}^{(2)}\exp ({\lambda }_{n}^{(2)}z)+{B}_{n}^{(2)}\exp (-{\lambda }_{n}^{(2)}z)]\,{\rm{for}}\,{\bf{r}}\in {\Omega }_{2},$$

12 $${V}_{3}({\bf{r}})={V}_{3,c}+\mathop{\sum }\limits_{n=1}^{+\infty }{J}_{0}({\lambda }_{n}^{(3)}\rho )[{A}_{n}^{(3)}\exp ({\lambda }_{n}^{(3)}z)+{B}_{n}^{(3)}\exp (-{\lambda }_{n}^{(3)}z)]\,{\rm{for}}\,{\bf{r}}\in {\Omega }_{3},$$

13 $${V}_{4}({\bf{r}})={V}_{4,c}+\mathop{\sum }\limits_{n=1}^{+\infty }{J}_{0}({\lambda }_{n}^{(4)}\rho )[{A}_{n}^{(4)}\exp ({\lambda }_{n}^{(4)}z)+{B}_{n}^{(4)}\exp (-{\lambda }_{n}^{(4)}z)]\,{\rm{for}}\,{\bf{r}}\in {\Omega }_{4},$$

and

14 $${V}_{m}({\bf{r}})={V}_{m,c}+\mathop{\sum }\limits_{n=1}^{+\infty }{J}_{0}({\lambda }_{n}^{(m)}\rho )[{A}_{n}^{(m)}\exp ({\lambda }_{n}^{(m)}z)+{B}_{n}^{(m)}\exp (-{\lambda }_{n}^{(m)}z)]\,{\rm{for}}\,{\bf{r}}\in {\Omega }_{m},$$

implying harmonic–type potential functions in terms of Bessel functions of zeroth order *J*_0_^25^, whereas ${V}_{j,c}$ with $j=1,2,3,4,m$ are constant potentials. Obviously, the linearly independent Neumann solutions^23^ are excluded, since they become singular for $\rho =0$. The discrete parameters ${\lambda }_{n}^{(j)}$ for $j=1,2,3,4,m$ with $n\ge 1$, arising from the method of separation of variables to the Laplace’s equation, along with the unknown constant coefficients ${A}_{n}^{(j)}$ and ${B}_{n}^{(j)}$ for any value of $j=1,2,3,4,m$, have to be calculated from the appropriately fitted boundary conditions of the particular physical problem, those being

15 $$ \mbox{-} {\rm{Boundary}}\,\rho =0\,{\rm{and}}\,z\in [0,+\,\infty ):\,\frac{\partial {V}_{j}(0,\varphi ,z)}{\partial \rho }=0\,{\rm{for}}\,j=1,2,3,4,m$$

16 $$ \mbox{-} {\rm{Boundary}}\,\rho ={\rho }_{hd}\,{\rm{and}}\,z\in [0,{z}_{1}]:\,{V}_{1}({\rho }_{hd},\varphi ,z)=0$$

17 $${\rm{and}}\,z\in [{z}_{1},{z}_{2}]:\,\frac{\partial {V}_{2}({\rho }_{hd},\varphi ,z)}{\partial \rho }=0$$

18 $${\rm{and}}\,z\in [{z}_{2},{z}_{3}]:\,{V}_{3}({\rho }_{hd},\varphi ,z)={V}_{e}$$

19 $${\rm{and}}\,z\in [{z}_{3},{z}_{4}]:\,\frac{\partial {V}_{4}({\rho }_{hd},\varphi ,z)}{\partial \rho }=0$$

20 $${\rm{and}}\,z\in [{z}_{4},+\,\infty ):\,\frac{\partial {V}_{m}({\rho }_{hd},\varphi ,z)}{\partial \rho }=0$$

21 $$ \mbox{-} {\rm{Boundary}}\,z=0\,{\rm{and}}\,\rho \in [0,{\rho }_{hd}]:\,\frac{\partial {V}_{1}(\rho ,\varphi ,0)}{\partial z}=0$$

22 $$ \mbox{-} {\rm{Boundary}}\,z={z}_{1}\,{\rm{and}}\,\rho \in [0,{\rho }_{hd}]:\,{V}_{1}(\rho ,\varphi ,{z}_{1})={V}_{2}(\rho ,\varphi ,{z}_{1})$$

23 $$\frac{\partial {V}_{1}(\rho ,\varphi ,{z}_{1})}{\partial z}=\frac{\partial {V}_{2}(\rho ,\varphi ,{z}_{1})}{\partial z}$$

24 $$ \mbox{-} {\rm{Boundary}}\,z={z}_{2}\,{\rm{and}}\,\rho \in [0,{\rho }_{hd}]:\,{V}_{2}(\rho ,\varphi ,{z}_{2})={V}_{3}(\rho ,\varphi ,{z}_{2})$$

25 $$\frac{\partial {V}_{2}(\rho ,\varphi ,{z}_{2})}{\partial z}=\frac{\partial {V}_{3}(\rho ,\varphi ,{z}_{2})}{\partial z}$$

26 $$ \mbox{-} {\rm{Boundary}}\,z={z}_{3}\,{\rm{and}}\,\rho \in [0,{\rho }_{hd}]:\,{V}_{3}(\rho ,\varphi ,{z}_{3})={V}_{4}(\rho ,\varphi ,{z}_{3})$$

27 $$\frac{\partial {V}_{3}(\rho ,\varphi ,{z}_{3})}{\partial z}=\frac{\partial {V}_{4}(\rho ,\varphi ,{z}_{3})}{\partial z}$$

28 $$ \mbox{-} {\rm{Boundary}}\,z={z}_{4}\,{\rm{and}}\,\rho \in [0,{\rho }_{hd}]:\,{V}_{4}(\rho ,\varphi ,{z}_{4})={V}_{m}(\rho ,\varphi ,{z}_{4})$$

29 $$\frac{\partial {V}_{4}(\rho ,\varphi ,{z}_{4})}{\partial z}=\frac{\partial {V}_{m}(\rho ,\varphi ,{z}_{4})}{\partial z}$$

30 $$ \mbox{-} {\rm{Limit}}\,z\to +\,\infty \,{\rm{and}}\,\rho \in [0,{\rho }_{hd}]:\,\mathop{\mathrm{lim}}\limits_{z\to +\infty }{V}_{m}(\rho ,\varphi ,z)=0$$

that comprise either Dirichlet–type and Neumann–type conditions or classical continuity conditions between common boundaries with no charges present.

## Electrostatic Potential and Electric Field in the CAPP Jet Systems

Primarily, condition (15) is readily satisfied, since ${J}_{0}^{{\prime} }(0)=-\,{J}_{1}(0)=0$ according to the definition of the Bessel’s functions^25^. In the sequel, the limiting condition (30), applied on the relative field (14), forces us to calculate

31 $${V}_{m,c}=0\,{\rm{and}}\,{A}_{n}^{(m)}=0\,{\rm{with}}\,n\ge 1$$

therefore, the latter becomes

32 $${V}_{m}({\bf{r}})=\mathop{\sum }\limits_{n=1}^{+\infty }{B}_{n}^{(m)}{J}_{0}({\lambda }_{n}^{(m)}\rho )\exp (-{\lambda }_{n}^{(m)}z)\,{\rm{for}}\,{\bf{r}}\in {\Omega }_{m}.$$

Next, symmetry condition (21), reinforced on the electrostatic potential field (10), reads

33 $$\mathop{\sum }\limits_{n=1}^{+\infty }{\lambda }_{n}^{(1)}({A}_{n}^{(1)}-{B}_{n}^{(1)}){J}_{0}({\lambda }_{n}^{(1)}\rho )=0\,{\rm{for}}\,{\rm{every}}\,\rho \in [0,{\rho }_{hd}],$$

which reveals that

34 $${A}_{n}^{(1)}-{B}_{n}^{(1)}=0\,{\rm{or}}\,{B}_{n}^{(1)}={A}_{n}^{(1)}\,{\rm{with}}\,n\ge 1$$

and, consequently, the implicated field (10) is rewritten as

35 $${V}_{1}({\bf{r}})={V}_{1,c}+\mathop{\sum }\limits_{n=1}^{+\infty }{C}_{n}^{(1)}{J}_{0}({\lambda }_{n}^{(1)}\rho )\cosh ({\lambda }_{n}^{(1)}z)\,{\rm{for}}\,{\bf{r}}\in {\Omega }_{g},$$

whereas ${C}_{n}^{(1)}\equiv 2{A}_{n}^{(1)}$ with $n\ge 1$ is a new constant coefficient. Our next task includes the manipulation of boundary conditions (16)–(20), which in view of (35), (11)–(13) and (32), they give

36 $${V}_{1,c}+\mathop{\sum }\limits_{n=1}^{+\infty }{C}_{n}^{(1)}{J}_{0}({\lambda }_{n}^{(1)}{\rho }_{hd})\cosh ({\lambda }_{n}^{(1)}z)=0\,{\rm{for}}\,{\rm{every}}\,z\in [0,{z}_{1}],$$

37 $$\mathop{\sum }\limits_{n=1}^{+\infty }{\lambda }_{n}^{(2)}{J}_{0}^{{\prime} }({\lambda }_{n}^{(2)}{\rho }_{hd})[{A}_{n}^{(2)}\exp ({\lambda }_{n}^{(2)}z)+{B}_{n}^{(2)}\exp (-{\lambda }_{n}^{(2)}z)]=0\,{\rm{for}}\,{\rm{every}}\,z\in [{z}_{1},{z}_{2}],$$

38 $${V}_{3,c}+\mathop{\sum }\limits_{n=1}^{+\infty }{J}_{0}({\lambda }_{n}^{(3)}{\rho }_{hd})[{A}_{n}^{(3)}\exp ({\lambda }_{n}^{(3)}z)+{B}_{n}^{(3)}\exp (-{\lambda }_{n}^{(3)}z)]={V}_{e}\,{\rm{for}}\,{\rm{every}}\,z\in [{z}_{2},{z}_{3}],$$

39 $$\mathop{\sum }\limits_{n=1}^{+\infty }{\lambda }_{n}^{(4)}{J}_{0}^{{\prime} }({\lambda }_{n}^{(4)}{\rho }_{hd})[{A}_{n}^{(4)}\exp ({\lambda }_{n}^{(4)}z)+{B}_{n}^{(4)}\exp (-{\lambda }_{n}^{(4)}z)]=0\,{\rm{for}}\,{\rm{every}}\,z\in [{z}_{3},{z}_{4}]$$

and

40 $$\mathop{\sum }\limits_{n=1}^{+\infty }{B}_{n}^{(m)}{\lambda }_{n}^{(m)}{J}_{0}^{{\prime} }({\lambda }_{n}^{(m)}{\rho }_{hd})\exp (-{\lambda }_{n}^{(m)}z)=0\,{\rm{for}}\,{\rm{every}}\,z\in [{z}_{4},+\infty ),$$

respectively. A direct result of (36) and (38) is that

41 $${V}_{1,c}=0\,{\rm{and}}\,{V}_{3,c}={V}_{e},$$

while in order to avoid trivial zero potential fields and since ${J}_{0}^{{\prime} }(x)=-\,{J}_{1}(x)$ for the different arguments $x\equiv {\lambda }_{n}^{(2)}{\rho }_{hd}$, $x\equiv {\lambda }_{n}^{(4)}{\rho }_{hd}$ and $x\equiv {\lambda }_{n}^{(m)}{\rho }_{hd}$, we are obliged to evaluate the corresponding involved separation constants as

42 $${\lambda }_{n}^{(1)}={\lambda }_{n}^{(3)}=\frac{{r}_{n}}{{\rho }_{hd}}\,{\rm{and}}\,{\lambda }_{n}^{(2)}={\lambda }_{n}^{(4)}={\lambda }_{n}^{(m)}=\frac{{s}_{n}}{{\rho }_{hd}}\,{\rm{with}}\,n\ge 1,$$

in terms of the roots of the Bessel’s function of zeroth (*r*_*n*_) and first (*s*_*n*_) order, that is ${J}_{0}({r}_{n})=0$ and ${J}_{1}({s}_{n})=0$. By virtue of (41), (42) and presuming ${V}_{2,c}={V}_{4,c}=0$ without loss of generality, the electrostatic potentials (35), (11)–(13) and (32) yield

43 $${V}_{1}({\bf{r}})=\frac{1}{2}\mathop{\sum }\limits_{n=1}^{+\infty }{D}_{n}^{(1)}{J}_{0}\left({r}_{n}\frac{\rho }{{\rho }_{hd}}\right)\left[\exp \left({r}_{n}\frac{z-{z}_{1}}{{\rho }_{hd}}\right)+\exp \left(-{r}_{n}\frac{z+{z}_{1}}{{\rho }_{hd}}\right)\right]\,{\rm{for}}\,{\bf{r}}\in {\Omega }_{1},$$

44 $${V}_{2}({\bf{r}})=\mathop{\sum }\limits_{n=1}^{+\infty }{J}_{0}\left({s}_{n}\frac{\rho }{{\rho }_{hd}}\right)\left[{C}_{n}^{(2)}\exp \left({s}_{n}\frac{z-{z}_{2}}{{\rho }_{hd}}\right)+{D}_{n}^{(2)}\exp \left(-{s}_{n}\frac{z-{z}_{1}}{{\rho }_{hd}}\right)\right]\,{\rm{for}}\,{\bf{r}}\in {\Omega }_{2},$$

45 $${V}_{3}({\bf{r}})={V}_{e}+\mathop{\sum }\limits_{n=1}^{+\infty }{J}_{0}\left({r}_{n}\frac{\rho }{{\rho }_{hd}}\right)\left[{C}_{n}^{(3)}\exp \left({r}_{n}\frac{z-{z}_{3}}{{\rho }_{hd}}\right)+{D}_{n}^{(3)}\exp \left(-{r}_{n}\frac{z-{z}_{2}}{{\rho }_{hd}}\right)\right]\,{\rm{for}}\,{\bf{r}}\in {\Omega }_{3},$$

46 $${V}_{4}({\bf{r}})=\mathop{\sum }\limits_{n=1}^{+\infty }{J}_{0}\left({s}_{n}\frac{\rho }{{\rho }_{hd}}\right)\left[{C}_{n}^{(4)}\exp \left({s}_{n}\frac{z-{z}_{4}}{{\rho }_{hd}}\right)+{D}_{n}^{(4)}\exp \left(-{s}_{n}\frac{z-{z}_{3}}{{\rho }_{hd}}\right)\right]\,{\rm{for}}\,{\bf{r}}\in {\Omega }_{4}$$

and

47 $${V}_{m}({\bf{r}})=\mathop{\sum }\limits_{n=1}^{+\infty }{D}_{n}^{(m)}{J}_{0}\left({s}_{n}\frac{\rho }{{\rho }_{hd}}\right)\exp \left(-{s}_{n}\frac{z-{z}_{4}}{{\rho }_{hd}}\right)\,{\rm{for}}\,{\bf{r}}\in {\Omega }_{m},$$

wherein the exponential calibration into (43)–(47) has been made for analytical and numerical convenience purposes, by denoting ${D}_{n}^{(1)}\equiv {C}_{n}^{(1)}\exp ({r}_{n}{z}_{1}/{\rho }_{hd})$ and ${D}_{n}^{(m)}\equiv {B}_{n}^{(m)}\exp (-{s}_{n}{z}_{4}/{\rho }_{hd})$, ${C}_{n}^{(k)}\equiv {A}_{n}^{(k)}\exp ({q}_{n}{z}_{k}/{\rho }_{hd})$ and ${D}_{n}^{(k)}\equiv {B}_{n}^{(k)}\exp (-{q}_{n}{z}_{k-1}/{\rho }_{hd})$ for $k=2,3,4$ (note ${q}_{n}={s}_{n}$ for $k=2,4$, while ${q}_{n}={r}_{n}$ for $k=3$) the new coefficients, entered within (43)–(47).

On the other hand, the manipulation of the rest of the transmission in the *z*–direction conditions (22)–(29), initially require the proper representation of the constant potential *V*_*e*_ via a series expansion in terms of Bessel eigensolutions and to this end we adopt the following expansion^25^ within the domain $\rho \in [0,{\rho }_{hd}]$, i.e.

48 $${V}_{e}=2{V}_{e}\mathop{\sum }\limits_{n=1}^{+\infty }\frac{1}{{r}_{n}{J}_{1}({r}_{n})}{J}_{0}\left({r}_{n}\frac{\rho }{{\rho }_{hd}}\right)\,{\rm{for}}\,{\rm{any}}\,\rho \in [0,{\rho }_{hd}],$$

which comprises the Fourier–Bessel expansion of ${V}_{e}$. Proceeding, conditions (22)–(29) embody potentials (43)–(47) with (48) in such a way so as to arrive at

49 $$\begin{array}{ll} & \frac{1}{2}\mathop{\sum }\limits_{n=1}^{+\infty }{D}_{n}^{(1)}{J}_{0}\left({r}_{n}\frac{\rho }{{\rho }_{hd}}\right)\left[1+\exp \left(-{r}_{n}\frac{2{z}_{1}}{{\rho }_{hd}}\right)\right]\\ = & \mathop{\sum }\limits_{n=1}^{+\infty }{J}_{0}\left({s}_{n}\frac{\rho }{{\rho }_{hd}}\right)\left[{C}_{n}^{(2)}\,\exp \left(-{s}_{n}\frac{{z}_{2}-{z}_{1}}{{\rho }_{hd}}\right)+{D}_{n}^{(2)}\right],\,\rho \in [0,{\rho }_{hd}]\end{array}$$

and

50 $$\begin{array}{ll} & \frac{1}{2}\mathop{\sum }\limits_{n=1}^{+\infty }{r}_{n}{D}_{n}^{(1)}{J}_{0}\left({r}_{n}\frac{\rho }{{\rho }_{hd}}\right)\left[1-\exp \left(-{r}_{n}\frac{2{z}_{1}}{{\rho }_{hd}}\right)\right]\\ = & \mathop{\sum }\limits_{n=1}^{+\infty }{s}_{n}{J}_{0}\left({s}_{n}\frac{\rho }{{\rho }_{hd}}\right)\left[{C}_{n}^{(2)}\exp \left(-{s}_{n}\frac{{z}_{2}-{z}_{1}}{{\rho }_{hd}}\right)-{D}_{n}^{(2)}\right],\,\rho \in [0,{\rho }_{hd}],\end{array}$$

while

51 $$\begin{array}{ll} & \mathop{\sum }\limits_{n=1}^{+\infty }{J}_{0}\left({s}_{n}\frac{\rho }{{\rho }_{hd}}\right)\left[{C}_{n}^{(2)}+{D}_{n}^{(2)}\,\exp \left(-{s}_{n}\frac{{z}_{2}-{z}_{1}}{{\rho }_{hd}}\right)\right]\\ = & \mathop{\sum }\limits_{n=1}^{+\infty }{J}_{0}\left({r}_{n}\frac{\rho }{{\rho }_{hd}}\right)\left[\frac{2{V}_{e}}{{r}_{n}{J}_{1}({r}_{n})}+{C}_{n}^{(3)}\,\exp \left(-{r}_{n}\frac{{z}_{3}-{z}_{2}}{{\rho }_{hd}}\right)+{D}_{n}^{(3)}\right]\,{\rm{for}}\,\rho \in [0,{\rho }_{hd}]\end{array}$$

and

52 $$\begin{array}{ll} & \mathop{\sum }\limits_{n=1}^{+\infty }{s}_{n}{J}_{0}\left({s}_{n}\frac{\rho }{{\rho }_{hd}}\right)\left[{C}_{n}^{(2)}-{D}_{n}^{(2)}\,\exp \left(-{s}_{n}\frac{{z}_{2}-{z}_{1}}{{\rho }_{hd}}\right)\right]\\ = & \mathop{\sum }\limits_{n=1}^{+\infty }{r}_{n}{J}_{0}\left({r}_{n}\frac{\rho }{{\rho }_{hd}}\right)\left[{C}_{n}^{(3)}\,\exp \left(-{r}_{n}\frac{{z}_{3}-{z}_{2}}{{\rho }_{hd}}\right)-{D}_{n}^{(3)}\right]\,{\rm{for}}\,\rho \in [0,{\rho }_{hd}],\end{array}$$

whilst

53 $$\begin{array}{ll} & \mathop{\sum }\limits_{n=1}^{+\infty }{J}_{0}\left({r}_{n}\frac{\rho }{{\rho }_{hd}}\right)\left[\frac{2{V}_{e}}{{r}_{n}{J}_{1}({r}_{n})}+{C}_{n}^{(3)}+{D}_{n}^{(3)}\exp \left(-{r}_{n}\frac{{z}_{3}-{z}_{2}}{{\rho }_{hd}}\right)\right]\\ = & \mathop{\sum }\limits_{n=1}^{+\infty }{J}_{0}\left({s}_{n}\frac{\rho }{{\rho }_{hd}}\right)\left[{C}_{n}^{(4)}\,\exp \left(-{s}_{n}\frac{{z}_{4}-{z}_{3}}{{\rho }_{hd}}\right)+{D}_{n}^{(4)}\right]\,{\rm{for}}\,\rho \in [0,{\rho }_{hd}]\end{array}$$

and

54 $$\begin{array}{ll} & \mathop{\sum }\limits_{n=1}^{+\infty }{r}_{n}{J}_{0}\left({r}_{n}\frac{\rho }{{\rho }_{hd}}\right)\left[{C}_{n}^{(3)}-{D}_{n}^{(3)}\exp \left(-{r}_{n}\frac{{z}_{3}-{z}_{2}}{{\rho }_{hd}}\right)\right]\\ = & \mathop{\sum }\limits_{n=1}^{+\infty }{s}_{n}{J}_{0}\left({s}_{n}\frac{\rho }{{\rho }_{hd}}\right)\left[{C}_{n}^{(4)}\exp \left(-{s}_{n}\frac{{z}_{4}-{z}_{3}}{{\rho }_{hd}}\right)-{D}_{n}^{(4)}\right]\,{\rm{for}}\,\rho \in [0,{\rho }_{hd}],\end{array}$$

while

55 $$\mathop{\sum }\limits_{n=1}^{+\infty }{J}_{0}\left({s}_{n}\frac{\rho }{{\rho }_{hd}}\right)\left[{C}_{n}^{(4)}+{D}_{n}^{(4)}\exp \left(-{s}_{n}\frac{{z}_{4}-{z}_{3}}{{\rho }_{hd}}\right)\right]=\mathop{\sum }\limits_{n=1}^{+\infty }{D}_{n}^{(m)}{J}_{0}\left({s}_{n}\frac{\rho }{{\rho }_{hd}}\right)\,{\rm{for}}\,\rho \in [0,{\rho }_{hd}]$$

and

56 $$\mathop{\sum }\limits_{n=1}^{+\infty }{s}_{n}{J}_{0}\left({s}_{n}\frac{\rho }{{\rho }_{hd}}\right)\left[{C}_{n}^{(4)}-{D}_{n}^{(4)}\exp \left(-{s}_{n}\frac{{z}_{4}-{z}_{3}}{{\rho }_{hd}}\right)\right]=-\,\mathop{\sum }\limits_{n=1}^{+\infty }{s}_{n}{D}_{n}^{(m)}{J}_{0}\left({s}_{n}\frac{\rho }{{\rho }_{hd}}\right)\,{\rm{for}}\,\rho \in [0,{\rho }_{hd}]$$

respectively. Among above relations, only (55) and (56) can be treated straightforwardly by imposing basic orthogonality arguments of the Bessel functions of zeroth order^25^, implying

57 $${C}_{n}^{(4)}+{D}_{n}^{(4)}\exp \left(-{s}_{n}\frac{{z}_{4}-{z}_{3}}{{\rho }_{hd}}\right)={D}_{n}^{(m)}\,{\rm{with}}\,n\ge 1$$

and

58 $${C}_{n}^{(4)}-{D}_{n}^{(4)}\exp \left(-{s}_{n}\frac{{z}_{4}-{z}_{3}}{{\rho }_{hd}}\right)=-\,{D}_{n}^{(m)}\,{\rm{with}}\,n\ge 1,$$

which provide us with the set of solutions

59 $${C}_{n}^{(4)}=0\,{\rm{and}}\,{D}_{n}^{(m)}={D}_{n}^{(4)}\exp \left(-{s}_{n}\frac{{z}_{4}-{z}_{3}}{{\rho }_{hd}}\right)\,{\rm{with}}\,n\ge 1,$$

leading to the fact that potential fields (46) and (47) coincide to

60 $${V}_{4}({\bf{r}})\equiv {V}_{m}({\bf{r}})=\mathop{\sum }\limits_{n=1}^{+\infty }{D}_{n}^{(4)}{J}_{0}\left({s}_{n}\frac{\rho }{{\rho }_{hd}}\right)\exp \left(-{s}_{n}\frac{z-{z}_{3}}{{\rho }_{hd}}\right)\,{\rm{for}}\,{\bf{r}}\in {\Omega }_{4}\cup {\Omega }_{m},$$

whereas the section of sets (6) and (7) is

61 $${\Omega }_{4}\cup {\Omega }_{m}=\{{\bf{r}}\equiv (\rho ,\varphi ,z)\in {{\mathbb{R}}}^{3}:\rho \in (0,{\rho }_{hd}),\varphi \in [0,2\pi ),z\in ({z}_{3},+\,\infty )\},$$

representing an important result that secures the matching of the fields due to the same Neumann condition for $\rho ={\rho }_{hd}$. The transition conditions (49)–(54) involve different arguments of Bessel functions of zeroth order and they are treated in view of the orthogonality relation

62 $${\int }_{0}^{{\rho }_{hd}}\rho {J}_{0}\left({r}_{n}\frac{\rho }{{\rho }_{hd}}\right){J}_{0}\left({r}_{n{\prime} }\frac{\rho }{{\rho }_{hd}}\right)d\rho ={\delta }_{nn{\prime} }\frac{{[{\rho }_{hd}{J}_{1}({r}_{n})]}^{2}}{2}\,{\rm{with}}\,n,n{\prime} \ge 1$$

and the trivial integral

63 $${\int }_{0}^{{\rho }_{hd}}\rho {J}_{0}\left({s}_{n}\frac{\rho }{{\rho }_{hd}}\right){J}_{0}\left({r}_{n{\prime} }\frac{\rho }{{\rho }_{hd}}\right)d\rho =-\,\frac{{\rho }_{hd}^{2}{r}_{n{\prime} }{J}_{0}({s}_{n}){J}_{1}({r}_{n{\prime} })}{{s}_{n}^{2}-{r}_{n{\prime} }^{2}}\,{\rm{with}}\,n,n{\prime} \ge 1.$$

Specifically, we multiply both sides of conditions (49)–(54) by $\rho {J}_{0}\left({r}_{n{\prime} }\frac{\rho }{{\rho }_{hd}}\right)d\rho$ with $n{\prime} \ge 1$, we integrate over the interval $[0,{\rho }_{hd}]$ and we make use of (59), as well as the notations

64 $${D}_{n/n{\prime} }=\frac{2}{{r}_{n{\prime} }^{2}-{s}_{n}^{2}}\frac{{J}_{0}({s}_{n})}{{J}_{1}({r}_{n{\prime} })}\,{\rm{and}}\,{V}_{n{\prime} }=\frac{2{V}_{e}}{{r}_{n{\prime} }{J}_{1}({r}_{n{\prime} })}\,{\rm{with}}\,n,n{\prime} \ge 1$$

and the conveniently defined function

65 $$f(z\,;{q}_{n})=\exp \left({q}_{n}\frac{z}{{\rho }_{hd}}\right),\,{\rm{where}}\,{q}_{n}=\{\begin{array}{c}{r}_{n}\\ {s}_{n}\end{array}\,{\rm{with}}\,n\ge 1,$$

to obtain the following relations for the constant coefficients, in view of (62) and (63), i.e.

66 $$[1+f(-2{z}_{1};{r}_{n{\prime} })]{D}_{n{\prime} }^{(1)}-2{r}_{n{\prime} }\mathop{\sum }\limits_{n=1}^{+\infty }{D}_{n/n{\prime} }[f({z}_{1}-{z}_{2};{s}_{n}){C}_{n}^{(2)}+{D}_{n}^{(2)}]=0\,{\rm{with}}\,n{\prime} \ge 1,$$

67 $$[1-f(-2{z}_{1};{r}_{n{\prime} })]{D}_{n{\prime} }^{(1)}-2\mathop{\sum }\limits_{n=1}^{+\infty }{s}_{n}{D}_{n/n{\prime} }[f({z}_{1}-{z}_{2};{s}_{n}){C}_{n}^{(2)}-{D}_{n}^{(2)}]=0\,{\rm{with}}\,n{\prime} \ge 1,$$

68 $${r}_{n{\prime} }\mathop{\sum }\limits_{n=1}^{+\infty }{D}_{n/n{\prime} }[{C}_{n}^{(2)}+f({z}_{1}-{z}_{2};{s}_{n}){D}_{n}^{(2)}]-f({z}_{2}-{z}_{3};{r}_{n{\prime} }){C}_{n{\prime} }^{(3)}-{D}_{n{\prime} }^{(3)}={V}_{n{\prime} }\,{\rm{with}}\,n{\prime} \ge 1,$$

69 $$\mathop{\sum }\limits_{n=1}^{+\infty }{s}_{n}{D}_{n/n{\prime} }[{C}_{n}^{(2)}-f({z}_{1}-{z}_{2};{s}_{n}){D}_{n}^{(2)}]-f({z}_{2}-{z}_{3};{r}_{n{\prime} }){C}_{n{\prime} }^{(3)}+{D}_{n{\prime} }^{(3)}=0\,{\rm{with}}\,n{\prime} \ge 1,$$

70 $${C}_{n{\prime} }^{(3)}+f({z}_{2}-{z}_{3};{r}_{n{\prime} }){D}_{n{\prime} }^{(3)}-{r}_{n{\prime} }\mathop{\sum }\limits_{n=1}^{+\infty }{D}_{n/n{\prime} }{D}_{n}^{(4)}=-\,{V}_{n{\prime} }\,{\rm{with}}\,n{\prime} \ge 1,$$

71 $${C}_{n{\prime} }^{(3)}-f({z}_{2}-{z}_{3};{r}_{n{\prime} }){D}_{n{\prime} }^{(3)}+\mathop{\sum }\limits_{n=1}^{+\infty }{s}_{n}{D}_{n/n{\prime} }{D}_{n}^{(4)}=0\,{\rm{with}}\,n{\prime} \ge 1.$$

System (66)–(71) stands for an infinite linear system of six sets of algebraic equations, including the six constant coefficients ${D}_{n{\prime} }^{(1)}$, ${C}_{n}^{(2)}$, ${D}_{n}^{(2)}$, ${C}_{n{\prime} }^{(3)}$, ${D}_{n{\prime} }^{(3)}$ and ${D}_{n}^{(4)}$ with $n,n{\prime} \ge 1$, which is handled with cut–off techniques in order to obtain quadratic systems, whenever the number of sets *n*′ ≥ 1 of relations (66)–(71) coincides with the number of terms *n* ≥ 1 of the infinite series, that is $n{\prime} \cong 1,2,\mathrm{..}.,N$ and $\mathop{\sum }\limits_{n=1}^{+\infty }(\cdots )\cong \mathop{\sum }\limits_{n=1}^{N}(\cdots )$, concerning (66)–(71). Notice that the specified value $N\in {{\mathbb{N}}}^{\ast }$ is conveniently chosen so as to obtain the order of the desired accuracy of the numerical implementation for the evaluation of the unknowns. Doing so, we have to solve systems of type 6 × 6, 12 × 12, …, 6*N* × 6*N*, thus, defining the *N* × *N* blocks of matrixes, each one of type 6 × 6 (a total 6*N* × 6*N* matrix) of the coefficients of the unknowns as72as well as denoting the vector $6N\times 1$ of the unknown coefficients via

73 $${{\boldsymbol{x}}}_{n}={[\begin{array}{cccccccc}\cdots  & {D}_{n}^{(1)} & {C}_{n}^{(2)} & {D}_{n}^{(2)} & {C}_{n}^{(3)} & {D}_{n}^{(3)} & {D}_{n}^{(4)} & \cdots \end{array}]}^{{\rm{{\rm T}}}}$$

and incorporating the vector $6N\times 1$ of the known constants through

74 $${{\boldsymbol{b}}}_{n}={[\begin{array}{cccccccc}\cdots  & 0 & 0 & {V}_{n} & 0 & -{V}_{n} & 0 & \cdots \end{array}]}^{{\rm{{\rm T}}}},$$

then the block of systems (66)–(71) can be written in matrix form with a unique solution as

75 $${{\bf{A}}}_{n}\,{{\boldsymbol{x}}}_{n}={{\boldsymbol{b}}}_{n}\Rightarrow {{\boldsymbol{x}}}_{n}={{\bf{A}}}_{n}^{-1}{{\boldsymbol{b}}}_{n}\,{\rm{for}}\,{\rm{every}}\,n\cong 1,2,\,\mathrm{..}.,\,N\,{\rm{with}}\,N\in {{\mathbb{N}}}^{\ast }$$

since the determinant of **A**_*n*_ is not zero, hence the inverse matrix exists. The linear systems (75) with (72)–(74) for the evaluation of the constant coefficients ${D}_{n}^{(1)}$, ${C}_{n}^{(2)}$, ${D}_{n}^{(2)}$, ${C}_{n}^{(3)}$, ${D}_{n}^{(3)}$ and ${D}_{n}^{(4)}$ for $n=1,2,\mathrm{..}.,N$ and in terms of (64) and (65) were solved using MATLAB. The number of terms, taken $N=4000$ for all cases, was adequate to ensure a smooth transition of the solution between the domains, in the results area, which extends radially from the cylindrical symmetry axis to the internal surface of the dielectric tube. It is noted that closer to the upper boundary of the solution domain, i.e. to the electrode (see Fig. 1(b,d)), more terms would be required in order to obtain a smooth curve for the potential fields.

Once the unknown constant coefficients are evaluated, then the solution of the particular electrostatic potential boundary value problem is given by

76 $${V}_{1}({\bf{r}})=\frac{1}{2}\mathop{\sum }\limits_{n=1}^{+\infty }{D}_{n}^{(1)}{J}_{0}\left({r}_{n}\frac{\rho }{{\rho }_{hd}}\right)[f(z-{z}_{1};{r}_{n})+f(-z-{z}_{1};{r}_{n})]\,{\rm{for}}\,{\bf{r}}\in {\Omega }_{1},$$

77 $${V}_{2}({\bf{r}})=\mathop{\sum }\limits_{n=1}^{+\infty }{J}_{0}\left({s}_{n}\frac{\rho }{{\rho }_{hd}}\right)[{C}_{n}^{(2)}f(z-{z}_{2};{s}_{n})+{D}_{n}^{(2)}f({z}_{1}-z;{s}_{n})]{\rm{for}}\,{\bf{r}}\in {\Omega }_{2},$$

78 $${V}_{3}({\bf{r}})={V}_{e}+\mathop{\sum }\limits_{n=1}^{+\infty }{J}_{0}\left({r}_{n}\frac{\rho }{{\rho }_{hd}}\right)[{C}_{n}^{(3)}f(z-{z}_{3};{r}_{n})+{D}_{n}^{(3)}f({z}_{2}-z;{r}_{n})]{\rm{for}}\,{\bf{r}}\in {\Omega }_{3},$$

and

79 $${V}_{4}({\bf{r}})\equiv {V}_{m}({\bf{r}})=\mathop{\sum }\limits_{n=1}^{+\infty }{D}_{n}^{(4)}{J}_{0}\left({s}_{n}\frac{\rho }{{\rho }_{hd}}\right)f({z}_{3}-z;{s}_{n})\,{\rm{for}}\,{\bf{r}}\in {\Omega }_{4}\cup {\Omega }_{m},$$

provided definition (65). By virtue of (8) and (76)–(79), the electric field in terms of the obtained electrostatic potential at any point ${\bf{r}}(\rho ,\varphi ,z)$ is given by the concrete relationship

80 $${\bf{E}}(\rho ,\varphi ,z)=-\,\nabla V(\rho ,\varphi ,z)\,{\rm{for}}\,{\rm{any}}\,\rho \in [0,{\rho }_{hd}],\,\varphi \in [0,2\pi )\,{\rm{and}}\,z\in [0,+\,\infty ],$$

whereas

81 $$\begin{array}{rcl}V(\rho ,\varphi ,z) & = & H({z}_{1}-z){V}_{1}(\rho ,\varphi ,z)+[H({z}_{2}-z)-H({z}_{1}-z)]{V}_{2}(\rho ,\varphi ,z)\\  &  & +\,[H({z}_{3}-z)-H({z}_{2}-z)]{V}_{3}(\rho ,\varphi ,z)\\  &  & +\,[H({z}_{4}-z)-H({z}_{3}-z)]{V}_{4}(\rho ,\varphi ,z)\\  &  & +\,H(z-{z}_{4}){V}_{m}(\rho ,\varphi ,z),\end{array}$$

in view of the Heaviside step function *H*(*x*) = 1 for *x* > 0, otherwise zero and at $\rho \in [0,{\rho }_{hd}]$, $\varphi \in [0,2\pi )$ and $z\in [0,+\,\infty ]$, which ends our analytical model solution in the present standard CAPP jet reactor systems.

## Numerical Implementation Results and Discussion

### Single electrode reactor (two–region problem/Fig. 1(a) and 1(c))

**Figure 3:**
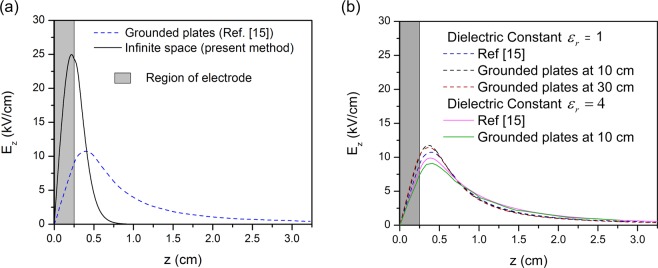
(**a**) Axial electric field distribution for a single electrode reactor. The blue dashed line shows the curve from figure No. 1 of Jánský and Bourdon^15^, where grounded planes are placed at a finite length from the electrode (i.e. 10 cm from the edge of the driven ring, at *z* = 10.25 cm). The black solid line shows the present analytical solution with zero–potential boundary condition at infinity. The grey area corresponds to the region inside the annular electrode (half of the electrode is considered with symmetry boundary conditions). (**b**) Comparison of numerical solutions with dielectric constants ${\varepsilon }_{r}=1$ and ${\varepsilon }_{r}=4$, and different distances of the grounded planes, as they are copied from Jánský and Bourdon^15^ or obtained by our own numerical runs of their equations (agreement with AIP Publishing – license number: 4753080774327).

The first reactor that is considered is based on the work of Jánský and Bourdon^15^. The electric field is created by a single electrode wrapped around a dielectric tube with internal radius and wall thickness of 1.6 mm. The length of the electrode is 5 mm (in the present calculations the half–length is considered with symmetry boundary condition) and it is biased with a constant applied voltage of 12 kV. In the numerical simulation of Jánský and Bourdon^15^, grounded planes were considered at the edges of the computational domain, in order to ensure that the potential decreases down to zero far from the electrode. Specifically, on the *z*–axis, grounded planes were placed at 10 cm from left and right edges of the driven ring, i.e. at *z* = 10 cm with reference to Fig. 3 (see ref. ^15^ for details on the solution domain). In the present analysis, the zero potential boundary condition is set at the boundary of the semi–infinite domain, so there is no grounded electrode. It is noted that in both cases, the tube permittivity is approximated close to 1, similar to that of the gas flowing through the tube (i.e. helium), according to the assumption discussed above. The differences in the axial component of the electric field along the symmetry axis are shown in Fig. 3(a), where the grey area corresponds to the region inside the annular electrode. The dashed blue curve was copied from figure No. 1 of Jánský and Bourdon^15^ and the solid black line depicts the results obtained from our analytical approach. The present calculations predict a much higher peak value (almost 2.5 times higher) and a much steeper decrease, compared to the numerical results with the grounded electrodes. The electric field in the present analytical solution is zero at an axial distance of 5 mm from the electrode, while in the results with the grounded plates the field extends farther than 30 mm from the electrode, although it is very weak. It must be noted that the main differences are not due to the distance between the grounded planes and the electrode. It can be verified that placing the grounded planes at longer distances from the electrode has an effect that cannot alone account for the differences on the axial electric field distribution. Indeed, to assess the effect of increasing the distance of the grounded plates in numerical solutions and the discrepancy due to the dielectric constant of the tube, we solved numerically the Laplace equation, using grounded planes at different distances from the electrode. The solution of the Laplace equation was conducted with the OpenFOAM C++ library^31^, using multi–region support. Figure 3(b) shows the comparison of these numerical solutions with the results of Jánský and Bourdon^15^ for two values of the dielectric constant and for grounded planes at different distances. The graphs support the above statement.

Consequently, the discrepancies between the numerical and the analytical results are synergistically attributed to: (i) The conventional choice to solve the mixed–type boundary value problems of our model (Laplace’s Eq. (9)) by applying cylindrical harmonic eigenfunctions only. (ii) The fact that the analytical model developed here is not expanded to the radial domain considered by Jánský and Bourdon^15^. (iii) The different modelling of the boundary conditions. Specifically, in the present analytical solution with the zero–potential boundary condition at infinity, the decay of the electric potential in the axial direction is exponential. Otherwise, if the zero–potential boundary condition is placed at a finite distance, though far from the electrode, via grounded planes, the decay of the electric potential is not necessarily purely exponential. (iv) The non–inclusion of the dielectric layer in analytical model. These points determine the prospective work that should be realized as a continuity of the present report.

**Figure 4:**
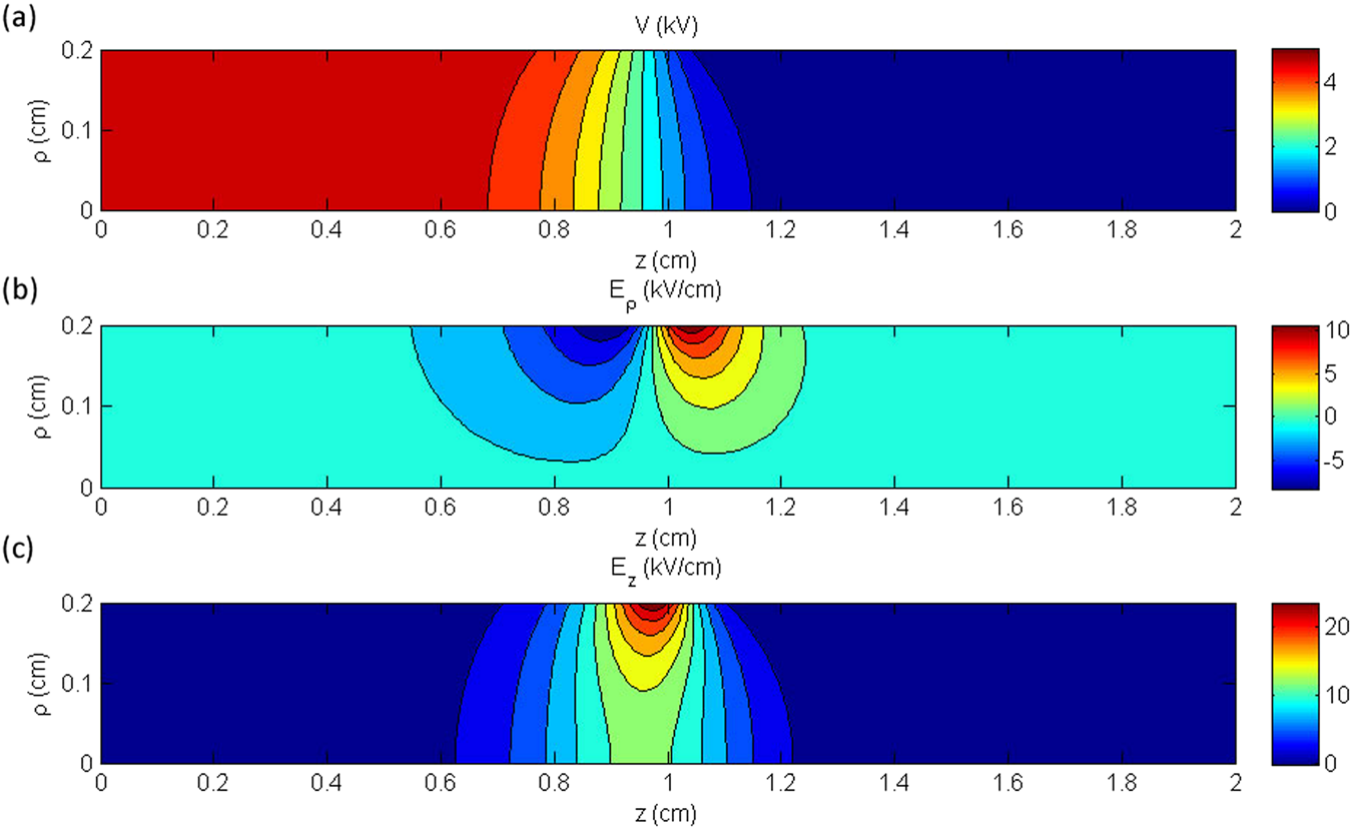
Contours of (**a**) the electrostatic potential (**b**) the electric field in the radial direction and (**c**) the electric field in the axial direction, for the single electrode reactor.

The second single electrode reactor is based on the experimental configuration reported recently by our group^13^ (modified in the sense that the grounded electrode has been removed). It consists of an annular electrode 20 mm in length and internal radius of 3 mm (see Fig. 1(a)), fixed around a dielectric tube of 1 mm wall thickness. The applied voltage is maintained here at +5 kV. This value has been selected according to our previous experiments, i.e. when the reactor of reference^13^ was driven by sinusoidal high voltage at 10 kHz, a peak–to–peak amplitude of around (2 × 7) kV was necessary to ignite the electrical discharge, whereas when the same reactor was driven by pulsed high voltage (2 kHz; rising/falling time ~100 ns; duty cycle 10%)^32^, a plateau of about +7 kV was the threshold for discharge ignition. Thus, the amplitude of +5 kV is here selected as a value being not far from a practical operating one but at the same time being low enough to prevent any space charge formation (electrostatic field case). The middle of the 20 mm electrode is positioned at axial coordinate *z* = 0 mm, where the symmetry conditions are imposed, so the electrode extends up to *z* = 10 mm. Figure 4 shows the contours of the electrostatic potential (Fig. 4(a)), the radial electric field (Fig. 4(b)) and the axial electric field (Fig. 4(c)) in the area inside the dielectric tube, following the analytical solution. The radius *ρ* of the plotted area ranges from 0 to 2 mm (i.e. the inner radius of the dielectric tube) and the axial distance ranges from *z* = 0 mm, which corresponds to the center of the electrode, to *z* = 20 mm, where the electrostatic potential and the electric field are close to zero. The results show that the potential drops very steeply and its value becomes negligible after approximately 2 mm from the end of the electrode. Concerning the electric field, it is observed that the axial component is approximately double the radial one, and both components decrease down to zero at a few mm distance from the electrode.

**Figure 5:**
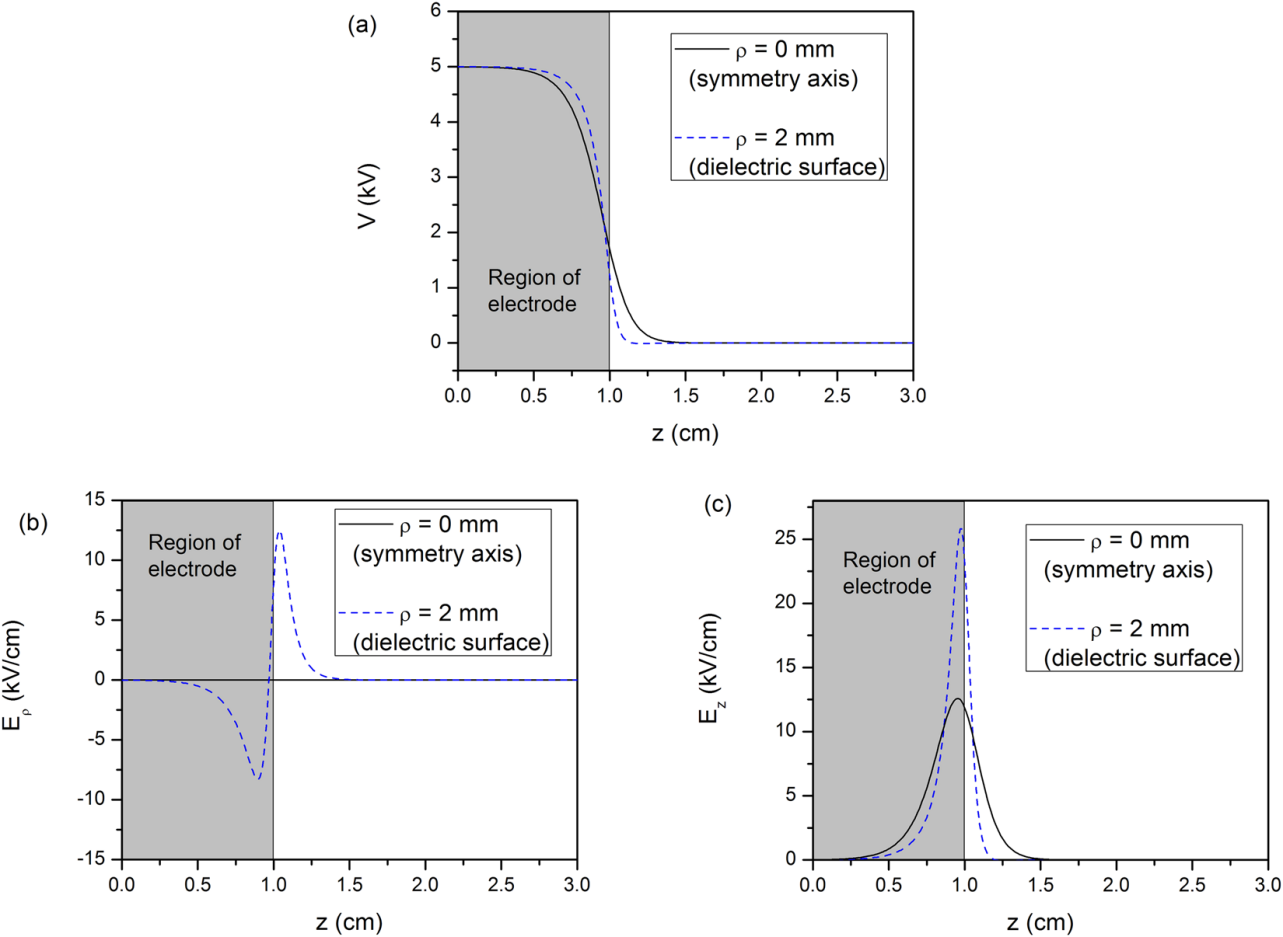
Axial distribution of (**a**) the electrostatic potential (**b**) the electric field in the radial direction and (**c**) the electric field in the axial direction at *ρ* = 0 mm (blue line) and *ρ* = 2 mm (blue dashed line). The negative sign in the values of (**b**) is qualitative and implies a direction opposite to the radial unit coordinate vector.

Figure 5 depicts the axial distribution of the electrostatic potential (Fig. 5(a)), the radial electric field (Fig. 5(b)) and the axial electric field (Fig. 5(c)), at two radii, one at the center of the cylindrical geometry *ρ* = 0 mm (black solid line) and one at the surface of the dielectric *ρ* = 2 mm (blues dashed line). The grey area corresponds to the region which is inside the annular electrode. The electrostatic potential is almost constant in that region, due to fact that the electrode is relatively long, compared to the one considered in the previous case (Fig. 3(a)). The electrostatic potential begins to decrease close to the edge of the electrode and, as expected, the drop is steeper close to the surface of the electrode (*ρ* = 2 mm) than farther from it, i.e. at the symmetry center–line. Beyond a distance of approximately 5 mm from the edge of the electrode, the potential becomes zero. The electric field plots (Fig. 5(b,c)) reveal that the there is a significant decrease of the electric field close to the symmetry axis. The radial component vanishes on the symmetry axis due to the symmetry boundary condition. The peak value of the axial electric field on the symmetry line is approximately half of the corresponding value on the surface of the electrode. Hence, it is inferred that the initiation of the plasma will occur on the surface of the electrode, where the electric field is stronger.

### Double electrode reactor (four–region problem/Fig. 1(b) and 1(d))

**Figure 6:**
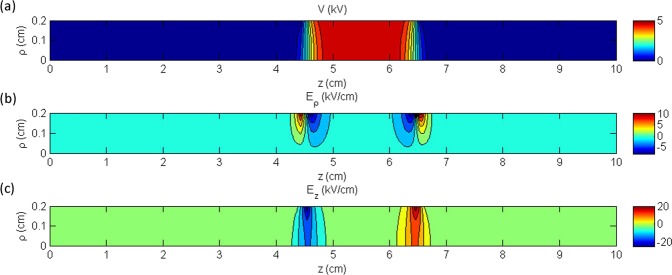
Contours of (**a**) the electrostatic potential (**b**) the electric field in the radial direction and (**c**) the electric field in the axial direction, for the double electrode reactor.

The double electrode reactor is based on the experimental configuration of our previous report^13^ and it is comprised of two annular electrodes with length 20 mm and internal radius 3 mm, which are positioned with a gap of 35 mm between them (see Fig. 1(b)). The thickness of the dielectric tube is 1 mm and the applied voltage is 5 kV. The middle of the grounded electrode is positioned at *z* = 0 mm, where the symmetry condition is imposed, and it extends up to *z* = 10 mm. The electrically biased electrode extends from *z* = 45 mm to *z* = 65 mm. In Fig. 6, the electrostatic potential (Fig. 6(a)), the radial electric field (Fig. 6(b)) and the axial electric field (Fig. 6(c)) inside the dielectric tube’s area depict the analytic solution. The radius *ρ* of the plotted area ranges from 0 to 2 mm (i.e. the inner radius of the dielectric tube) and the axial distance ranges from *z* = 0 mm (center of the grounded electrode) to *z* = 100 mm.

The results show that the potential decreases very rapidly, almost symmetrically, in both sides of the driven electrode. As in the single electrode case, the axial electric field is approximately double the radial one, but both components fade away after a few mm from the edge of the driven electrode. The region inside the grounded electrode, i.e. from *z* = 0 mm to *z* = 10 mm has zero potential and electric field, thus the only electrically active area is that around the driven electrode. This fact leads to the conclusion that the existence of a grounded electrode does not affect the electric field significantly, compared to the single electrode case.

**Figure 7:**
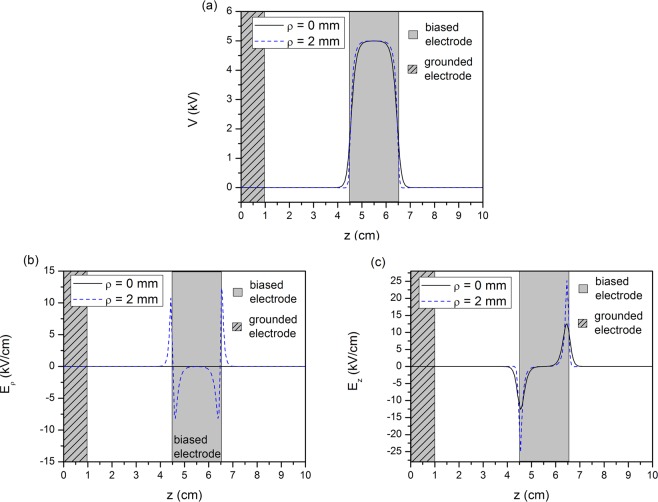
Axial distribution of (**a**) the electrostatic potential (**b**) the electric field in the radial direction and (**b**) the electric field in the axial direction at *ρ* = 0 mm (blue line) and *ρ* = 2 mm (blue dashed line). The negative sign in the values of (**b**) is qualitative and implies a direction opposite to the radial unit coordinate vector.

This observation can also be made from the plots of Fig. 7, which depict the axial distribution of the electrostatic potential (Fig. 7(a)), the radial electric field (Fig. 7(b)) and the axial electric field (Fig. 7(c)), at two radii, one at the center of the cylindrical geometry *ρ* = 0 mm (black solid line) and one at the surface of the dielectric *ρ* = 2 mm (blues dashed line). The grey line–filled area corresponds to the region which is inside the grounded electrode and the grey area without the line–filling corresponds to the region inside the driven electrode. It can be seen from all the figures that the electrostatic potential and the electric field have non–zero values only close to the driven electrode. The electrostatic potential does not have significant differences between the symmetry line at *ρ* = 0 mm and the dielectric surface at *ρ* = 2 mm. The electric fields components, however, are higher on the surface of the dielectric, similarly to the single electrode case. Additionally, the peak values of the electric field components are similar to the ones found in the single electrode case, which supports the notion that in the specific reactor configuration with the specific dimensions, the existence of a grounded electrode does not affect the electric field.

## Conclusions

In this contribution we have presented an analytical model for calculating the electrostatic potential and the electric field in plasma–jet reactors (CAPP jets). The solution is based on a multiple–region domain within the working gas that enables the calculation of the electric field in different reactor configurations, comprised of one or two electrodes with different sizes and dimensions. The contribution of the dielectric tube to the field variation is considered neglected. This is necessary in order to obtain the analytical solution and, although it may affect the electric field (especially its radial component) it is considered as a fair approximation, based on physical argumentation.

The mathematical modelling of the particular problem is focused on the solution of elliptic–type boundary value problems with either Dirichlet or Neumann boundary conditions, accompanied by the proper behavior of the fields at infinity, which approximates the area at the exit of the jet. Thus, every field potential is written in terms of harmonic cylindrical eigenmodes and three–dimensional expressions are derived as closed–type series formulae. Once the electrostatic potential is obtained within the entire domain of interest, the electric field is readily recovered and all cases are being numerically implemented and discussed, confirming the validity of the analytical approach.

Specifically, the results for the single electrode case have shown the important role of the location of the zero–potential boundary condition, in order to calculate the accurate electric field. The specific boundary condition is difficult to impose at infinity in numerical solutions, so the analytical solution presented in the present study is a useful approach for calculating the electric field in plasma simulations. Moreover, the results for the double–electrode reactor showed that for specific electrode configurations, the existence of a grounded electrode may not affect the electric field and consequently the conditions for the initiation of the plasma. Finally, the solution presented in the present paper could be a useful tool in the design of plasma–jet reactors for tailored applications. To this end, the present mathematical model must be enhanced by incorporating the effect of the dielectric tube, expanding the domain to radial distances approaching the zero–potential boundary condition, adding more terms in the present main analytical solution. Work under progress is set towards these directions.
